# Non-alcoholic fatty liver disease causally affects the brain cortical structure: a Mendelian randomization study

**DOI:** 10.3389/fnins.2023.1305624

**Published:** 2024-01-08

**Authors:** Yu-Kai Lin, Xin-Ran Cai, Jiang-Zhi Chen, Hai-Jie Hong, Kai Tu, Yan-Ling Chen, Qiang Du

**Affiliations:** ^1^Department of Hepatological Surgery and Fujian Institute of Hepatobiliary Surgery, Fujian Medical University Union Hospital, Fuzhou, China; ^2^Fujian Medical University Cancer Center, Fuzhou, China

**Keywords:** Mendelian randomization, non-alcoholic fatty liver disease, brain cortical structure, liver–brain axis, genome-wide association study

## Abstract

**Background:**

Reduced brain volume, impaired cognition, and possibly a range of psychoneurological disorders have been reported in patients with non-alcoholic fatty liver disease (NAFLD); however, no underlying cause has been specified. Here, Mendelian randomization (MR) was employed to determine the causative NAFLD effects on cortical structure.

**Methods:**

We used pooled-level data from FinnGen’s published genome-wide association study (GWAS) of NAFLD (1908 cases and 340,591 healthy controls), as well as published GWAS with NAFLD activity score (NAS) and fibrosis stage-associated SNPs as genetic tools, in addition to the Enigma Consortium data from 51,665 patients, were used to assess genetic susceptibility in relation to changes with cortical thickness (TH) and surface area (SA). A main estimate was made by means of inverse variance weighted (IVW), while heterogeneity and pleiotropy were detected using MR-Egger, weighted median, and MR Pleiotropy RESidual Sum and Outlier to perform a two-sample MR analysis.

**Results:**

At the global level, NAFLD reduced SA (beta = −586.72 mm^2^, se = 217.73, *p* = 0.007) and several changes in the cortical structure of the cerebral gyrus were found, with no detectable pleiotropy.

**Conclusion:**

NAFLD causally affects cortical structures, which supports the presence of an intricate liver–brain axis.

## Introduction

Non-alcoholic fatty liver disease (NAFLD) affects approximately 25% of the global population (Younossi et al., 2016), making it the most prevalent chronic liver disease worldwide. By 2030, it is expected to become the leading indication for liver transplantation (Byrne and Targher, 2015), posing a significant global health burden. In the past, researchers have traditionally defined non-alcoholic fatty liver disease (NAFLD) solely based on the pathological findings of liver tissue in affected patients, encompassing simple hepatic steatosis (fatty liver), known as non-alcoholic fatty liver (NAFL) and non-alcoholic steatohepatitis (NASH), in which hepatocytes are subjected to chronic inflammatory damage with fibrosis. In 2020, a group of international experts proposed an update to the definition, suggesting “metabolic dysfunction-associated fatty liver disease (MAFLD),” and achieved a consensus. This revised definition better aligns with the underlying mechanisms of this liver disease, significantly advancing both research and clinical practice in this field (Eslam et al., 2020). Studies have suggested that NAFLD patients have reduced brain volume with cognitive impairment (Filipovic et al., 2018) and have found that NAFLD patients are more likely to suffer from central nervous system disorders, such as depression, anxiety (Lee et al., 2013), bipolar disorder, schizophrenia (Jawad et al., 2022), and Alzheimer’s disease (Estrada et al., 2019), suggesting that the liver–brain axis exists. Nevertheless, the causal relationship between NAFLD and cortical structures remains ambiguous and difficult to determine.

Plan and operation of the Third National Health and Nutrition Examination Survey, 1988-94. Series 1: programs and collection procedures (1994) described poor memory and attention in patients with NAFLD who also exhibited deficits in visuospatial function and psychomotor speed (simple reaction time test, SRTT) (Seo et al., 2016). Previous observational studies have certain limitations, including small sample sizes that can cause uncertainty in the findings. Furthermore, confounding factors and causal inversions are inherent limitations of observational epidemiology.

Mendelian randomization (MR) is a powerful method that utilizes genetic variation to infer causality in different diseases. It randomly assigns genetic alleles, including alternative and reference alleles, from parents to offspring during meiosis, similar to randomized controlled trials (RCTs). This approach minimizes the confounding bias inherent in observational studies (Davey Smith and Hemani, 2014; Paternoster et al., 2017). In this study, we conducted a two-sample MR analysis to investigate the effects of NAFLD on cortical structure. We used summary statistics from the most recent published genome-wide association studies (GWASs) to address this question using single nucleotide polymorphisms (SNPs) associated with specific disease phenotypes as instrumental variables (IVs) (Burgess et al., 2013). This approach allowed us to avoid direct analysis of individual-level data, which can be time-consuming and resource-intensive.

To determine how NAFLD affects the structure of the brain cortex, the human brain cortical thickness (TH) and surface area (SA) were measured with MRI. To produce the MR estimations, three sets of parameters—NAFLD, NAFLD activity score (NAS), and fibrosis stage—were employed. Additionally, we conducted subgroup analysis based on various functional areas. New information about the potential presence of a liver–brain axis is revealed by our findings.

## Methods

### GWAS summary data for NAFLD, NAS, and fibrosis stages

The summary-level GWAS data correlated with NAFLD were obtained from our previous GWAS study of European-ancestry participants from the FinnGen (r8.finngen.fi). Individuals with ICD code [ICD-10 K76.0 “Fatty (change of) liver, not elsewhere classified,” Excl.: nonalcoholic steatohepatitis (K75.8)] were characterized as NAFLD cases, including 1908 cases and 340,591 controls. For the GWAS of NAS and Liver fibrosis stage were obtained from Namjou et al. (2019), who conducted a case-only GWA quantitative study across the electronic Medical Records and Genomics (eMERGE) Network, focusing solely on NAFLD cases. This study identified highly associated SNPs related to NAS score, fibrosis. The dataset included 235 cases of European ancestry (characteristics of the exposure GWAS summary data listed in Supplementary Table S1). NAS is calculated based on a Kleiner-designed histological characteristics scoring system, available liver enzymes, and histopathological grading to assess the severity of NAFLD (Kleiner et al., 2005).

### GWAS summary data for brain cortex SA and cortex TH

The ENIGMA consortium was established in 2009 to conduct a large-scale global neuroimaging genetics study aimed at identifying the genetic markers most closely associated with brain structure and function (Thompson et al., 2020). GWAS data related to brain cortical structure were obtained from Grasby et al. (2020) by extracting brain cortex SA and TH from whole-brain T1-weighted MRI scans of 51,665 individuals from 60 cohorts worldwide, who were predominantly of European origin (~94%), and processed using FreeSurfer software. Our study utilized meta-analysis results from only 33,992 participants of European origin (23,909 from 49 cohorts participating in the ENIGMA consortium and 10,083 from the UK Biobank) (Supplementary Table S2 provides individual cohort details). We explored the entire cerebral cortex using the Desikan–Killiany atlas of 34 regions to determine whether changes occur in different cortical functional areas of the brain in response to NAFLD (Desikan et al., 2006). The cortical SA and TH of these regions were pre-averaged between the two hemispheres and were globally weighted or not depending on whether the whole brain was considered or not. Our final MR analysis of NAFLD, NASH, and fibrosis stage yielded a total of 414 results at both global and local levels.

### Selection of genetic instruments

We introduced two additional sets of genetic instruments for NAFLD severity characteristics while determining the causal relationship between NAFLD on cortical structures and NAFLD severity characteristics including NAS and fibrosis stage. The three sets of index SNPs we used are as follows: (1) index SNPs for NAFLD, (2) index SNPs for NAS, and (3) index SNPs for the fibrosis stage (detailed in Supplementary Tables S3–S5). NAFLD is a chronic disease that is accompanied by liver function impairment. The clinical diagnosis of NAFLD is typically made by combining alanine aminotransferase (ALT)-based methods with non-invasive clinical parameters. NAS and fibrosis stage are indicators of the severity of NAFLD as measured by histologic assessment of the degree of steatosis, necroinflammatory lesions evident in NASH, and fibrosis (Pouwels et al., 2022). Higher activity scores and histologic staging indicate a more intense inflammatory response and fibrotic process as well as extensive impairment of liver function. Supplementary measurement of histopathological features is necessary for the diagnosis of NAFL, in addition to previous diagnosis by liver enzymes and imaging features.

We selected genetic instruments for two-sample MR analyses based on the following criteria: (1) at genome-wide significant levels associated with exposure, we used a relatively relaxed threshold (5 × 10^−6^) to obtain a sufficient number of SNPs, and (2) a linkage disequilibrium [LD] r2 of <0.01, within 5,000 kb distance.

Following the removal of cortical structure-related SNPs within an upper limit of 5×10^−8^, we utilized MR Pleiotropy RESidual Sum and Outlier (MR-PRESSO) tests to identify and remove any underlying outliers prior to each MR analysis. MR-PRESSO is an optimal method for detecting and removing outliers in MR analysis, requiring at least 50% of the instrumental variables to be effective and satisfy the Instrument Strength Independent of Direct Effect (InSIDE) assumption (Verbanck et al., 2018).

We evaluated the power of the SNPs using the F statistic (F = beta2/se2) for each SNP and calculated a general F statistic for all SNPs. The strengths of the three genetic instruments used for MR analysis were 36.57, 24.94, and 25.45. Both F statistics exceeded the empirical strength threshold of 10 (Pierce et al., 2011).

### Mendelian randomization analyses

After harmonization of the effect alleles across the GWASs of NAFLD, NAS, fibrosis stage, and brain cortical structure, we employed multiple MR approaches to estimate the causal effect of exposure on outcomes, including the inverse variance weighted (IVW), weighted median, and MR-Egger methods, which have different assumptions based on horizontal pleiotropy. The IVW method was used as the primary outcome (Burgess et al., 2013), as it provides the most precise estimate, although it assumes that all SNPs are valid instruments. The weighted median method can provide consistent estimates when more than 50% of the weight comes from valid instrument variants. MR-Egger regression can generate estimates even when all instrumental variables are null, provided that the InSIDE hypothesis holds, although the precision of the estimates may be low (Bowden et al., 2015). The MR-Egger is considered supportive when the effect estimates are consistent with the direction of the IVW method. If the estimates of these approaches were inconsistent in our study, we set a stricter instrument value of *p* threshold (Supplementary Table S6) (Ong and MacGregor, 2019). We also evaluated horizontal pleiotropy using the MR-Egger intercept test, a funnel plot, and a leave-one-out analysis for significant estimates. The MR-Egger intercept was used as an indicator for directional pleiotropy (*p* < 0.05 was considered as evidence of directional pleiotropy). Cochran’s Q test was also used to identify heterogeneity (*p* < 0.05 was considered as evidence of heterogeneity). Additionally, we corrected outliers in instrumental variables (IVs) using MR-PRESSO. Using the Single Nucleotide Polymorphisms Annotator internet tool, we additionally evaluated the pleiotropy with probable confounders.^1^ All MR analyses were performed using the TwoSampleMR (version 0.4.25) package in R (version 4.2.2).

## Results

A total of 18 SNPs strongly associated with NAFLD, 11 SNPs strongly associated with NAS, and 38 SNPs strongly associated with fibrosis stage were screened.

**Figure 1:**
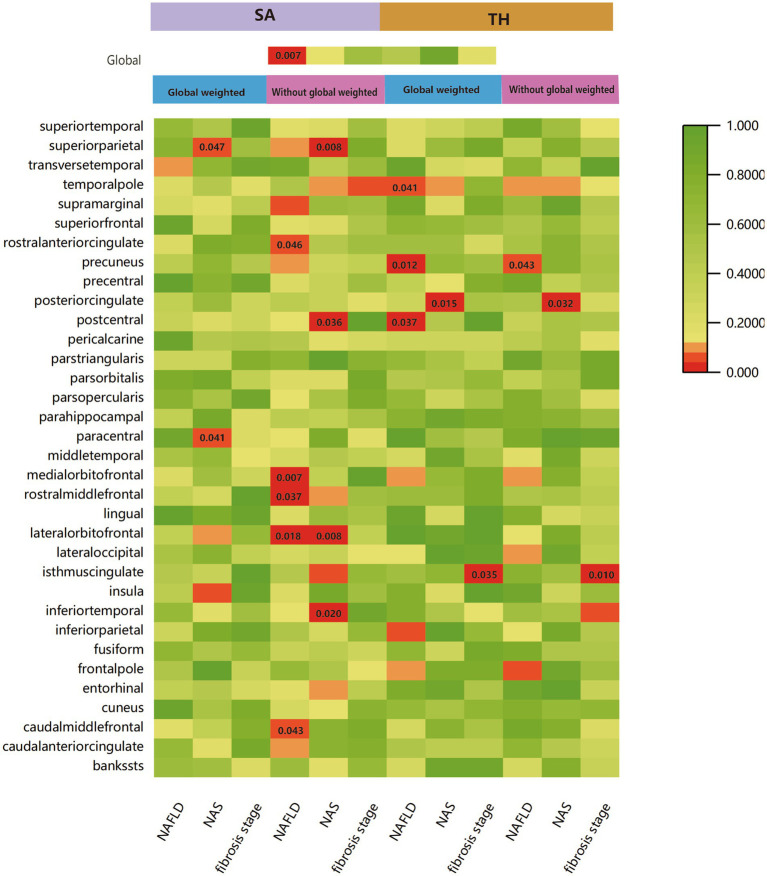
IVW estimates of cortical structure and 34 functional gyri from NAFLD, NAS, and fibrosis stages with and without global weighting. The different colors in the boxes represent the magnitude of the value of *p*.

**Figure 2:**
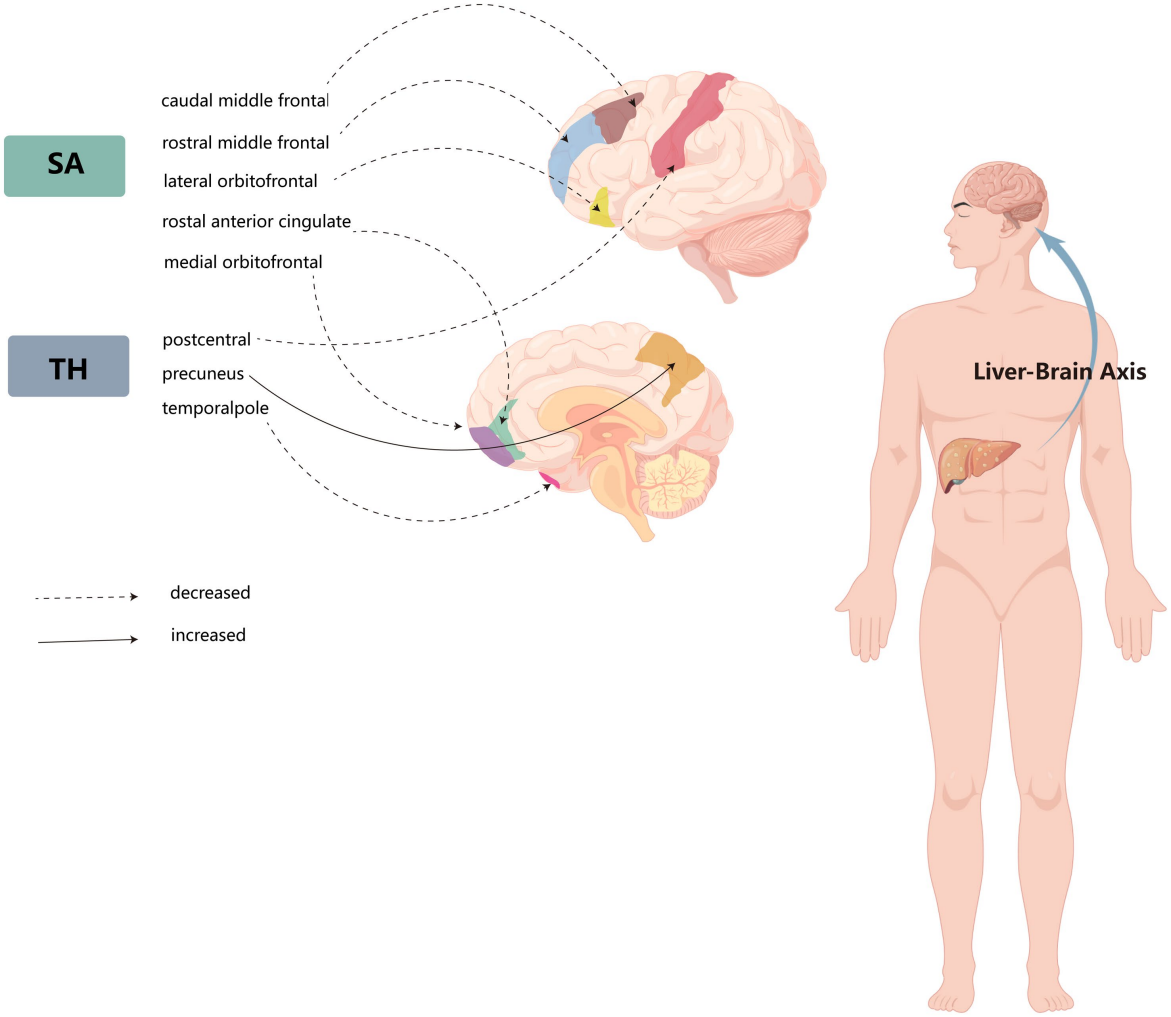
Schematic diagram of the liver–brain axis of the effects of non-alcoholic fatty liver disease on several cerebral gyrus cortices.

**Figure 3:**
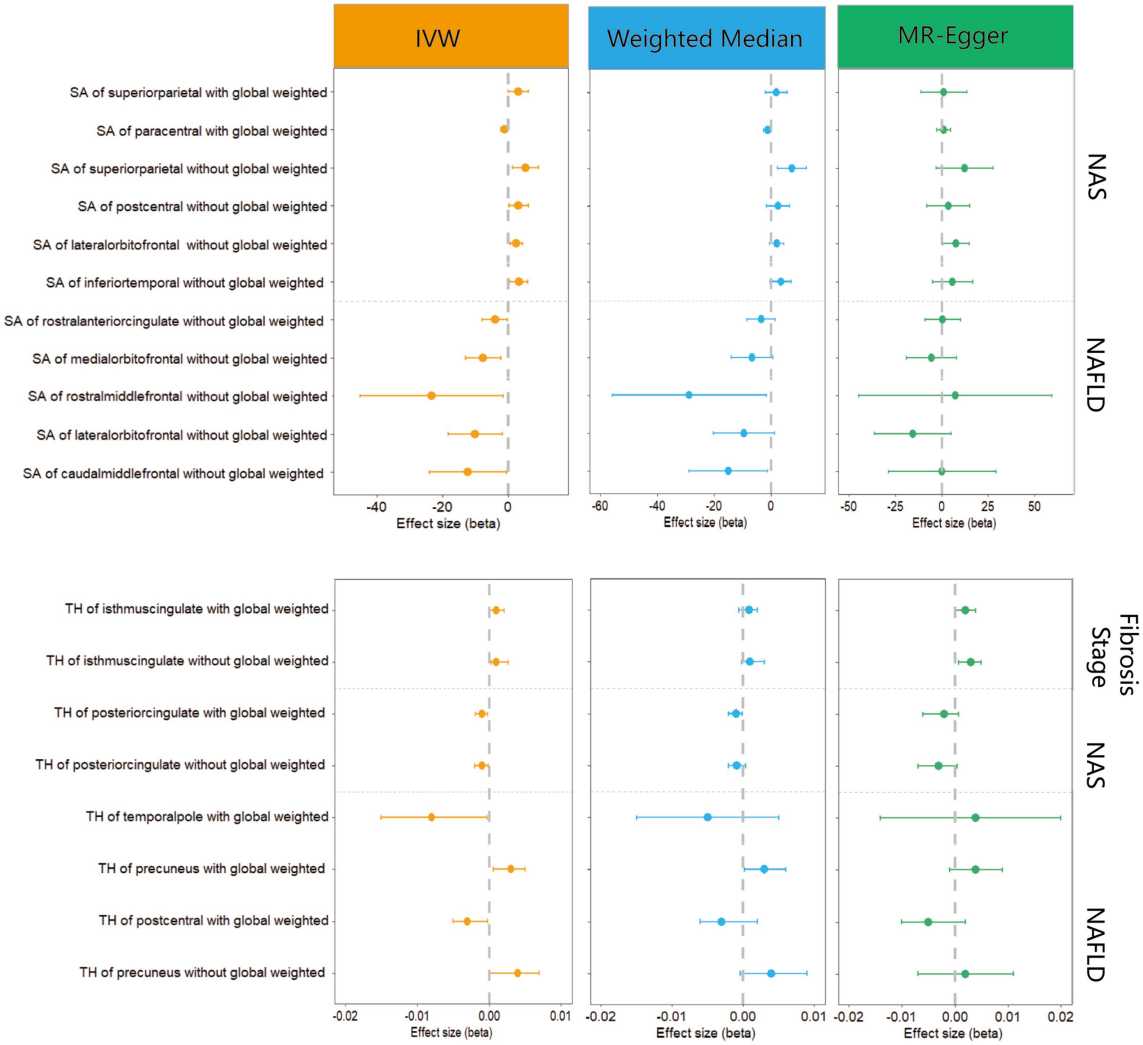
Forest plots of significant estimates identified with IVW.

We performed a comprehensive MR study to investigate the effects of genetically predicted NAFLD, NAS, and fibrosis stages on global SA/TH, as well as 34 functional gyri, with and without global weighting (Figure 1). Our analysis identified several significant gyri (Figure 2) affected by NAFLD using IVW, weighted median, and MR-Egger Methods, and all calculations are presented as forest plots (Figure 3). At the global level, NAFLD was initially not found to be significantly associated with SA (beta = −446.34 mm2, se = 267.62, *p* = 0.095). Heterogeneity was shown with a Cochran Q-derived value of *p* <0.05. MR-PRESSO also presented a similar result (value of *p* in the global heterogeneity test = 0.042). After removing one outlier (rs2745359), NAFLD was found to decrease SA (beta = −586.72 mm2, se = 217.73, *p* = 0.007) (Table 1; Supplementary Figure S1) but not TH (beta = 0.001 mm, se = 0.001, *p* = 0.456). NAS (beta_SA_ = 72.84, se_SA_ = 49.51, P_SA_ = 0.141; beta_TH_ = 5.06E-05, se_TH_ = 0.0003, P_TH_ = 0.883) and fibrosis stage (beta_SA_ = −24.63, se_SA_ = 43.74, P_SA_ = 0.573; beta_TH_ = 0.0004, se_TH_ = 0.0003, P_TH_ = 0.193) without apparent causality between the global SA and TH. Analysis at the functional region level, there were also several suggestive gyrus, NAFLD significantly decreased the SA of the caudal middle frontal, lateral orbitofrontal, rostral middle frontal, medial orbitofrontal, rostral anterior cingulate, without global weighting. NAFLD also decreased the TH of the postcentral, temporal pole, with global weighting. However, NAFLD increases the TH of precuneus with or without global weighting (Due to the unique genetic influences present in each brain region, the GWAS data for these areas were globally weighted across the entire brain to ensure comparability of changes in each region and to mitigate differences arising from genetic effects).

**Table 1:** Significant Mendelian randomization estimates from NAFLD, NAS, and fibrosis stage on the genetically predicted cortical structure.

Exposure	Outcome	IVW-derived value of *p*	Beta (95%Confidence intervals)	Cochran’s Q-derived value of *p*	MR-Egger intercept-derived value of *p*
NAFLD	Global SA	**0.007**	−586.72 mm2(−1013.47 to −159.97 mm2)	0.22	0.71
	SA of caudal middle frontal without global weighted	**0.043**	−12.17 mm2(−23.95 to −0.39 mm2)	0.07	0.38
	SA of lateral orbitofrontal without global weighted	**0.018**	−9.97 mm2(−18.23 to −1.71 mm2)	0.2	0.57
	SA of rostral middle frontal without global weighted	**0.037**	−23.24 mm2(−45.05 to −1.44 mm2)	0.19	0.23
	SA of medial orbitofrontal without global weighted	**0.007**	−7.53 mm2(−13.00 to −2.06 mm2)	0.43	0.74
	SA of rostral anterior cingulate without global weighted	**0.046**	−3.98 mm2(−7.89 to −0.07 mm2)	0.24	0.33
	TH of precuneus without global weighted	**0.043**	0.004 mm(0.0001 to 0.007 mm)	0.74	0.7
	TH of postcentral with global weighted	**0.037**	−0.003 mm(−0.005 to −0.0002)	0.31	0.51
	TH of precuneus with global weighted	**0.012**	0.003 mm(0.0006 to 0.005 mm)	0.91	0.68
	TH of temporal pole with global weighted	**0.041**	−0.008 mm(−0.015 to −0.0003 mm)	0.86	0.19
NAS	SA of inferior temporal without global weighted	**0.02**	3.30 mm2(0.52 to 6.09 mm2)	0.39	0.65
	SA of lateral orbitofrontal without global weighted	**0.008**	2.57 mm2(0.679 to 4.45 mm2)	0.6	0.19
	SA of postcentral without global weighted	**0.036**	3.25 mm2(0.218 to 6.29 mm2)	0.43	0.96
	SA of superior parietal without global weighted	**0.008**	5.43 mm2(1.43 to 9.43 mm2)	0.9	0.39
	SA of paracentral with global weighted	**0.041**	−1.04 mm2(−2.04 to −0.04 mm2)	0.67	0.29
	SA of superior parietal with global weighted	**0.048**	3.14 mm2(0.03 to 6.25 mm2)	0.18	0.76
	TH of posterior cingulate without global weighted	**0.032**	−0.001 mm(−0.002 to −0.00009 mm)	0.67	0.25
	TH of posterior cingulate with global weighted	**0.015**	−0.001 mm(−0.0019 to −0.0002 mm)	0.49	0.39
Fibrosis stage	TH of isthmus cingulate without global weighted	**0.01**	0.001 mm(0.0003 to 0.0026 mm)	0.63	0.16
	TH of isthmus cingulate with global weighted	**0.035**	0.001 mm(0.00008 to 0.0021 mm)	0.6	0.31

*p* < 0.05 suggests that it may be significant.

NAFLD, non-alcoholic fatty liver disease; NAS, NAFLD activity score; SA, cortical surface area; TH, cortical thickness; IVW, inverse variance weighted. Bold values suggest statistical significance.

In MR analysis of multiple NAFLD severity traits, higher NAS instead increased the SA of inferior temporal, lateral orbitofrontal, postcentral, and superior parietal with global weighting. Only the paracentral was decreasing SA with increasing NAS. Meanwhile, higher NAS decreased the TH of the posterior cingulate with or without global weighting. In another NAFLD severe traits, fibrotic state increased the SA of the isthmus cingulate with or without global weighting.

For both significant estimates, the MR-Egger intercept test and Cochran’s Q-derived *p*-values were > 0.05. In leave-one-out analyses, no abnormal SNPs were found that strongly violated the results. Moreover, the balanced funnel plot suggested the absence of horizontal pleiotropy (Supplementary Figures S2–S9).

## Discussion

This is the first large-scale MR study that reveals the causality of NAFLD on brain cortical structure. We utilized MR analysis to conduct a comprehensive evaluation of the relationship between genetically predicted NAFLD and two NAFLD severity characteristics as well as cortical structure in the present study. Our findings suggest that NAFLD may have a deleterious effect on the cerebral cortex, which is consistent with previous observational studies. The indication that NAFLD may act on the brain through relevant signaling pathways underscores the presence of a liver–brain axis (VanWagner et al., 2017), which has been increasingly recognized in recent years. Previous cross-sectional studies have demonstrated that patients with NAFLD are at a relatively high risk of cognitive impairment and central nervous system (CNS) disorders (Filipovic et al., 2018; Kjaergaard et al., 2021). Patients with neuropsychiatric disorders can be assessed by MRI for changes in brain structure, and the Montreal Cognitive Assessment (MoCA) is often used as a screening tool for cognitive impairment. However, the alteration of the cerebral cortex in NAFLD patients has been less reported in previous studies. The cerebral cortex is a crucial component of our complex cognitive abilities, and changes in the cortical structure have been genetically correlated with cognitive function, attention-deficit hyperactivity disorder, neuroticism, Parkinson’s disease insomnia, and depression (van Erp et al., 2018). Therefore, we postulate that there is a liver–brain axis in the human body that affects brain function through the liver–brain axis when there is a change or injury in the metabolism of the liver.

As NAFLD is a chronic disease, its associated complications often remain undetected. Moreover, the majority of NAFLD patients are middle-aged or elderly, and the cortical changes and cognitive impairment caused by NAFLD can be obscured by age-related brain aging changes. Therefore, it is imperative to investigate the causal mechanisms underlying the relationship between NAFLD and brain damage and to develop appropriate treatment plans for early detection and prevention. Such efforts will be crucial in mitigating the impact of NAFLD on cognitive function and overall health outcomes. Our study lays the groundwork for future research aimed at elucidating the mechanisms underlying the alterations in brain function and neuropsychiatric disorders associated with NAFLD.

This present study found that the entire cortical SA was decreased in patients with NAFLD, which may be related to the shrinkage of gray matter volumes throughout the brain (Filipovic et al., 2018). Previous studies have demonstrated a reduction in gray matter volume in patients with psychotic spectrum disorders (PSD) compared to healthy individuals, which may be attributed to a decrease in neural dendrites and a reduction in the number and size of neurons (Nazeri et al., 2020). Additionally, Nazeri et al. (2017) reported the presence of gray matter microstructural abnormalities and atrophy in PSD. Martina’s study points to cerebrovascular changes, neuroinflammation, and brain insulin resistance as the main pathological aspects linking NAFLD to cognitive impairment (Colognesi et al., 2020). Specifically, NAFLD may trigger gut microbiota ecological dysbiosis and urea cycle dysfunction, favoring ammonia accumulation and promoting further systemic inflammation with spread to the brain (Beydoun and Canas, 2022). Insulin resistance perpetuates adipose tissue inflammation and leads to the accumulation of ectopic lipids in various organs, including the liver, exacerbating liver damage and stimulating the production of large amounts of pro-inflammatory cytokines (Kjaergaard et al., 2021). Similarly, these inflammatory factors lead to cerebral atherosclerosis as well as cerebral microvascular hemodynamic dysfunction (Oni et al., 2013), causing reduced cerebral perfusion and hypoxia. These changes increase the risk of cortical atrophy and even asymptomatic subcortical infarction. One of the key neuroimmune factors is interleukin (IL)-6, which is upregulated in central nervous system disorders (Gadient and Otten, 1997) and has been implicated in conditions, such as schizophrenia, autism, depression, Parkinson’s disease, and Alzheimer’s disease (Garay and McAllister, 2010; Felger and Lotrich, 2013; Wei et al., 2013; Borsche et al., 2020). Other inflammatory mediators, such as CRP, IL6, TNFα, and VEGF, were observed to be differentially elevated in the peripheral blood of psychiatric patients. These distinct inflammatory mediators exhibited varying effects on the volume and thickness of different functional brain areas (Lizano et al., 2021).

MR analysis of 34 functional areas suggested an association with NAFLD in 8 gyri. Among them, the SA of the medial orbital front and the lateral orbital front were reduced to varying degrees. The orbitofrontal cortex (OFC) is located in the area of the brain directly above the eyes, and OFC is the primary neural mechanism for emotion production in humans and regulates social behavior (Bechara et al., 2000; Rolls et al., 2020). Abnormal structures or functions in the OFC have been observed in various psychiatric populations, including individuals with psychosis, obsessive-compulsive disorder, anxiety depression, substance use disorders, and antisocial disorders (Rudebeck and Rich, 2018). The SA of the caudal middle frontal gyrus was also reduced by NAFLD, and Nadia’s study showed a strong association between the caudal frontal gyrus and emotional dysregulation and physical aggression (Bounoua et al., 2022). The TH of the precuneus was increased by NAFLD in both globally weighted and unweighted. Considering its hidden location and the rarity of focal lesion studies, this cortical region usually gets little attention, but has extensive connectivity to subcortical structures (Cavanna and Trimble, 2006) and is selectively inactivated in many pathophysiological conditions (i.e., sleep, vegetative state, and drug-induced anesthesia) and neuropsychiatric disorders characterized by impaired consciousness (i.e., epilepsy, Alzheimer’s disease, and schizophrenia) (Cavanna, 2007). Whether NAFLD has a causal effect on the functional areas of the brain involved in the development of mental disorders can also be anticipated in future studies.

Weinstein et al. (2018) retrospectively analyzed 766 healthy middle-aged adults included in the study and found that NAFLD reduced total brain volume and accelerated brain aging. However, it is noteworthy that in the study of several severe characteristic indicators of NAFLD, mainly including NAS and fibrosis stage, it was observed that the surface area (SA) of specific functional areas, such as the inferior temporal, lateral orbitofrontal, postcentral, and superior parietal areas, increased with higher NAS scores. Similarly, we observed that isthmus cingulate cortex thickness in the fibrosis stage showed a tendency to increase with increasing fibrosis. This is different from the estimated expected logic, and we speculate that this may be related to the hypoxic and inflammatory response of the brain leading to cellular edema or compensatory hypertrophy of brain tissue.

To ensure a reliable MR analysis, three key assumptions must be met: (1) the association assumption, which requires a stable and robust correlation between instrumental variables and exposure factors; (2) the independence assumption, which necessitates that instrumental variables are independent of confounding factors that influence the “exposure-outcome” relationship; and (3) the exclusivity assumption, which stipulates that genetic factors affect the occurrence of outcomes solely through exposure factors. We selected exposures and results from different publicly available databases and did not find the same cohort overlap. In addition, we checked single nucleotide polymorphism annotators for the presence of SNPs associated with potential risk factors such as age, smoking, alcohol consumption, and cerebrovascular disease to avoid confounding factors.

This study also has limitations that need to be acknowledged. First, our NAFLD sample was obtained from the FinnGen, and it would be necessary to conduct a validation analysis using other recent GWAS databases to ensure the robustness of our MR analysis results. Second, in calculating the SA and TH for 34 regions in both hemispheres, we averaged the values, which may have overlooked functional and structural asymmetries. The extent of hemispheric differences may have obscured the final results for certain gyri.

Our findings offer new avenues for researchers to investigate the genetic variation in NAFLD and its impact on brain structure and function, particularly with respect to specific gyrus identified in our MRI analysis that have been previously linked to the development of neuropsychiatric disorders. However, further research is needed to fully understand the underlying mechanisms and to develop early interventions for the treatment of these unpredictable neuropsychiatric disorders in NAFLD patients.

## Conclusion

In conclusion, our MR analysis provides evidence of an association between NAFLD, NAS, fibrosis stages, and cortical structures, which may contribute to central nervous system (CNS) disease or dysfunction. These findings support the reality of a liver–brain axis by being consistent with previous animal experiments and observational studies. Our estimates suggest that NAFLD causally reduces global SA and changes in the cortical structures of several brain gyri. Therefore, routine brain MRI scans should be performed in patients with NAFLD as this may facilitate early detection and diagnosis of possible neuropsychiatric disorders.

## Data availability statement

The original contributions presented in the study are included in the article/Supplementary material, further inquiries can be directed to the corresponding authors.

## Author contributions

Y-KL: Conceptualization, Formal analysis, Methodology, Writing – original draft. XR-C: Data curation, Investigation, Writing – review & editing. J-ZC: Software, Validation, Writing – review & editing. H-JH: Data curation, Visualization, Writing – review & editing. KT: Software, Validation, Writing – review & editing. Y-LC: Project administration, Writing – review & editing. QD: Conceptualization, Funding acquisition, Project administration, Writing – review & editing.

## Footnotes

^1^ https://snipa.helmholtz-muenchen.de/snipa3/
