# Supplementary material for: Non-alcoholic fatty liver disease causally affects the brain cortical structure: a Mendelian randomization study
Source: Front Neurosci. 2024 Jan 8;17:1305624. doi: 10.3389/fnins.2023.1305624 (PMC10800802; doi:10.3389/fnins.2023.1305624)
Supplement: Supplementary file 1 [file Data_Sheet_1.docx]

Supplementary Information

**Non-alcoholic fatty liver disease Affects the Brain Cortical Structure: A Mendelian Randomization Study**

Yu-Kai Lin, MD^1,2^, Xin-Ran Cai, MD^1,2^, Jiang-Zhi Chen, MD, PhD ^1,2^, Hai-Jie Hong, MD, PhD ^1,2^, Kai Tu, MD^1,2^, Yan-Ling Chen, MD, PhD^1,2*^, Qiang Du, MD, PhD ^1,2*^

FigureS1. MR-Egger estimates of significant results from Non-alcoholic fatty liver disease on cortical SA and TH. (a)Scatter plots from genetically predicted Non-alcoholic fatty liver disease on global SA; (b)Leave-one-out plot from genetically predicted Non-alcoholic fatty liver disease on global SA; (c)Funnel plot from genetically predicted Non-alcoholic fatty liver disease on global SA.

**
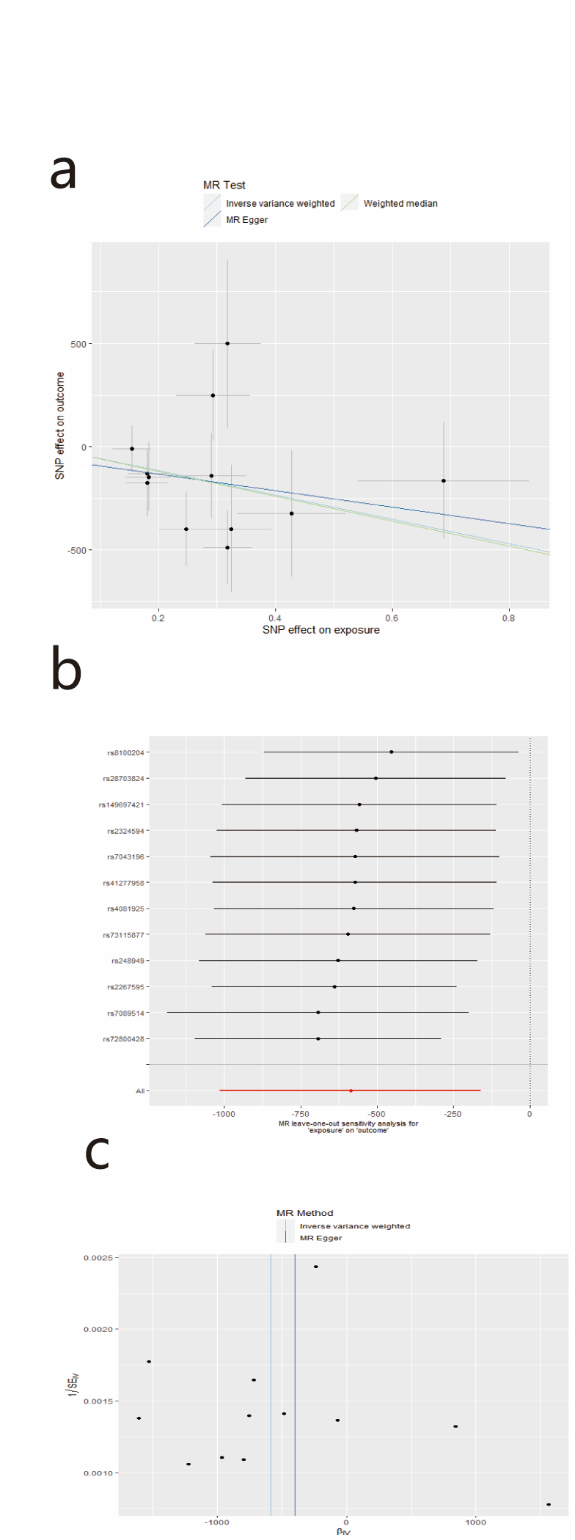
**

**Figure S2.** Scatter plots of significant estimates from genetically predicted Non-alcoholic fatty liver disease on (a) without global weighted SA of the caudalmiddlefrontal; (b) without global weighted SA of the lateralorbitofrontal; (c) without global weighted SA of the rostralmiddlefrontal; (d) without global weighted SA of medialorbitofrontal; (e) without global weighted SA of the rostralanteriorcingulate ; (f) without global weighted TH of the precuneus; (g) with global weighted TH of the postcentral; (h) with global weighted TH of the precuneus; (i) with global weighted TH of the temporalpole.
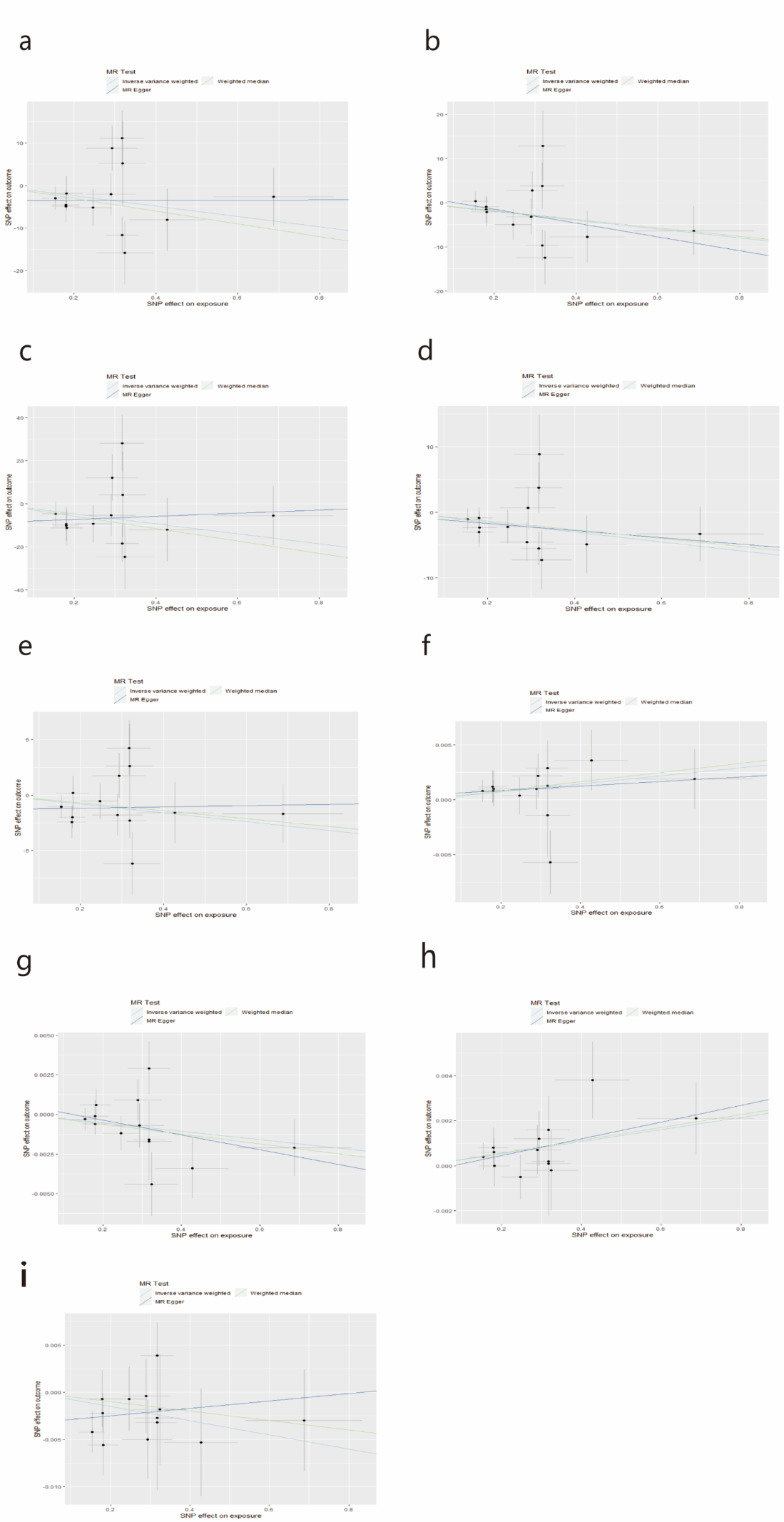


**Figure S3.** Leave-one-out plots of significant estimates from genetically predicted Non-alcoholic fatty liver disease on (a) without global weighted SA of the caudalmiddlefrontal; (b) without global weighted SA of the lateralorbitofrontal; (c) without global weighted SA of the rostralmiddlefrontal; (d) without global weighted SA of medialorbitofrontal; (e) without global weighted SA of the rostralanteriorcingulate ; (f) without global weighted TH of the precuneus; (g) with global weighted TH of the postcentral; (h) with global weighted TH of the precuneus; (i) with global weighted TH of the temporalpole.


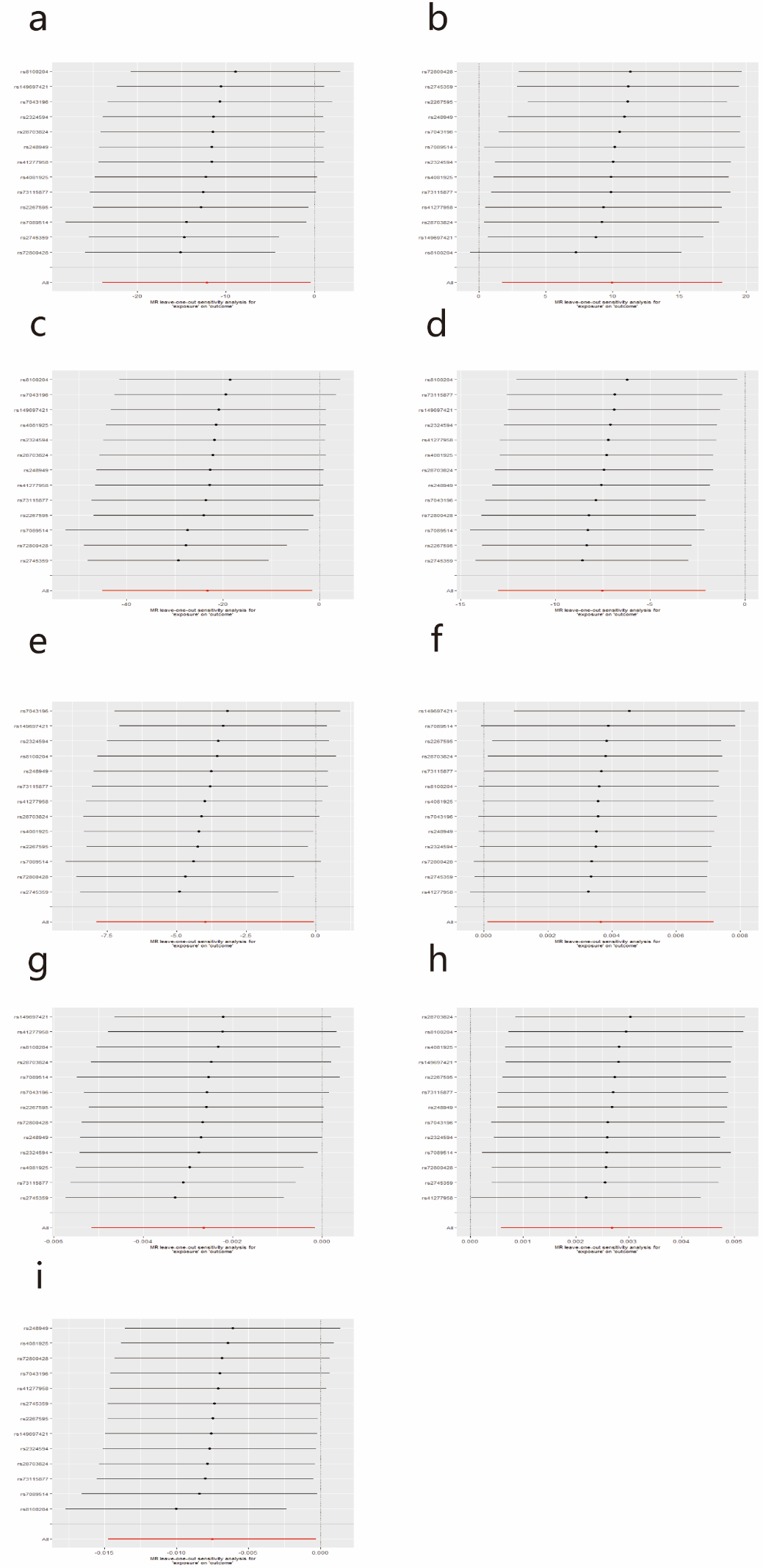


**Figure S4.** Funnel plots of significant estimates from genetically predicted Non-alcoholic fatty liver disease on (a) without global weighted SA of the caudalmiddlefrontal; (b) without global weighted SA of the lateralorbitofrontal; (c) without global weighted SA of the rostralmiddlefrontal; (d) without global weighted SA of medialorbitofrontal; (e) without global weighted SA of the rostralanteriorcingulate ; (f) without global weighted TH of the precuneus; (g) with global weighted TH of the postcentral; (h) with global weighted TH of the precuneus; (i) with global weighted TH of the temporalpole.

**
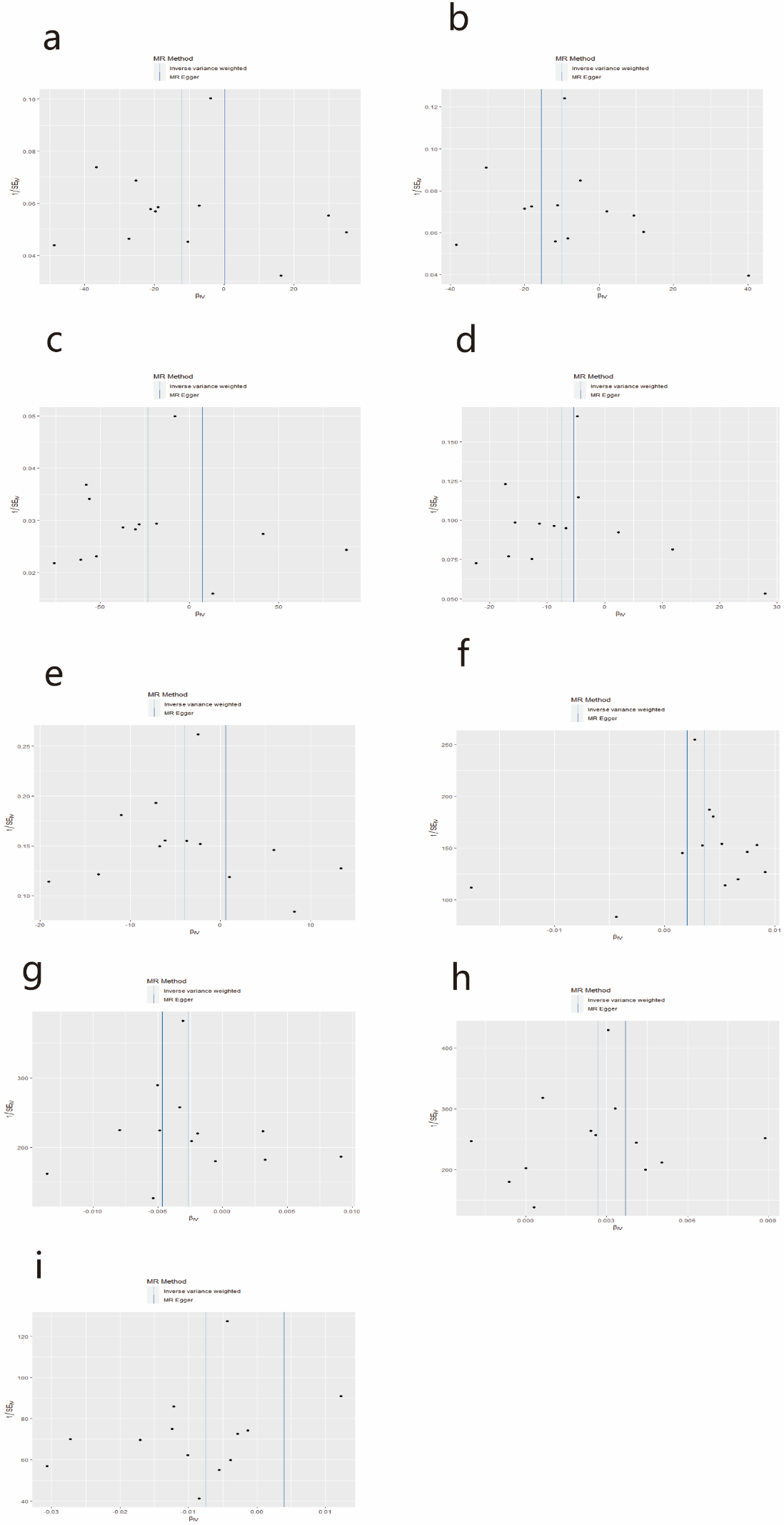
**

**Figure S5.** Scatter plots of significant estimates from genetically predicted NAS on (a) without global weighted SA of the inferiortemporal; (b) without global weighted SA of the lateralorbitofrontal; (c) without global weighted SA of the postcentral; (d) without global weighted SA of superiorparietal; (e) with global weighted SA of the paracentral ; (f) with global weighted SA of the superiorparietal; (g) without global weighted TH of the posteriorcingulate; (h) with global weighted TH of the posteriorcingulate.

**
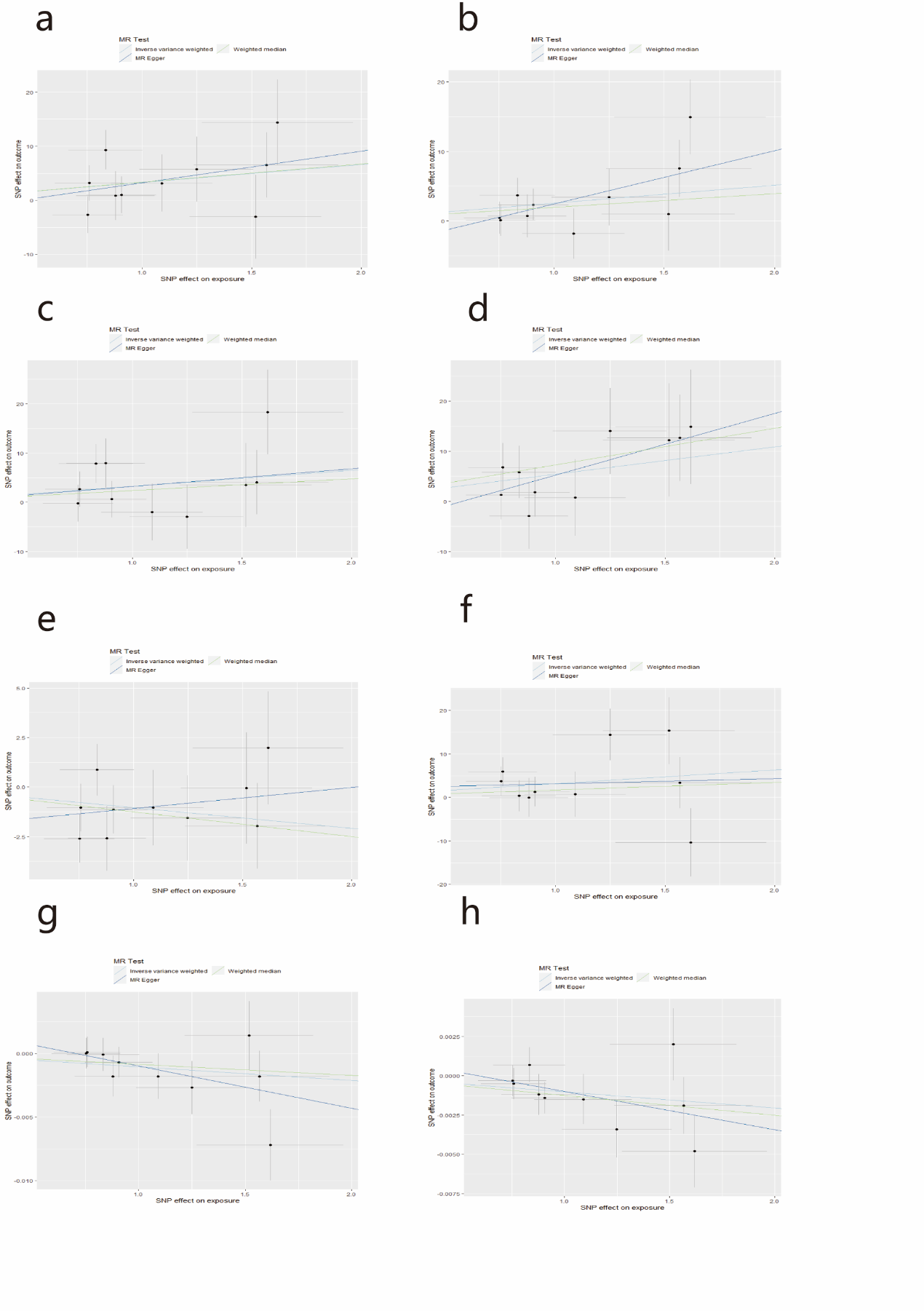
**

**Figure S6.** Leave-one-out plots of significant estimates from genetically predicted NAS on (a) without global weighted SA of the inferiortemporal; (b) without global weighted SA of the lateralorbitofrontal; (c) without global weighted SA of the postcentral; (d) without global weighted SA of superiorparietal; (e) with global weighted SA of the paracentral ; (f) with global weighted SA of the superiorparietal; (g) without global weighted TH of the posteriorcingulate; (h) with global weighted TH of the posteriorcingulate.

**
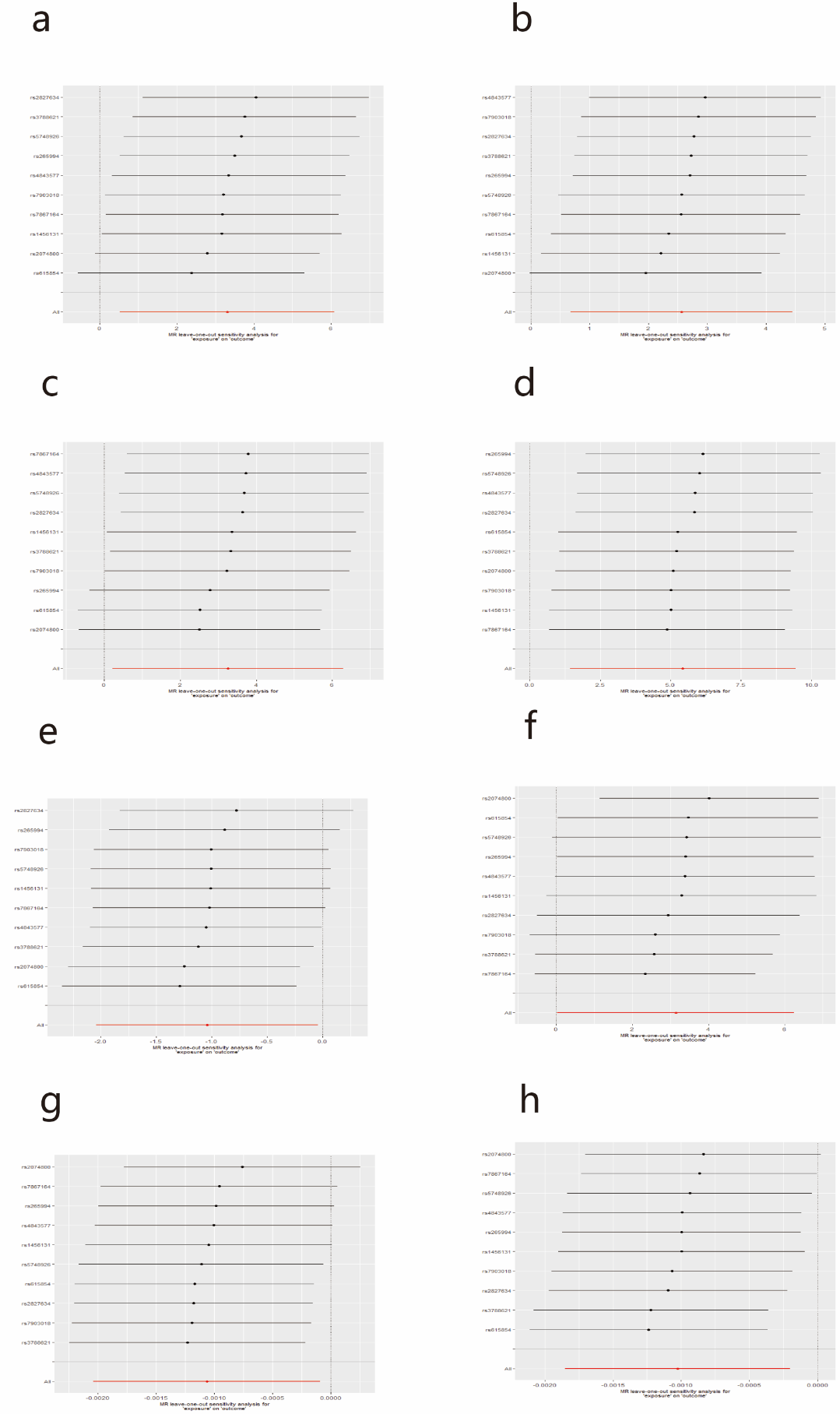
**

**Figure S6.** Funnel plots of significant estimates from genetically predicted NAS on (a) without global weighted SA of the inferiortemporal; (b) without global weighted SA of the lateralorbitofrontal; (c) without global weighted SA of the postcentral; (d) without global weighted SA of superiorparietal; (e) with global weighted SA of the paracentral ; (f) with global weighted SA of the superiorparietal; (g) without global weighted TH of the posteriorcingulate; (h) with global weighted TH of the posteriorcingulate.

**
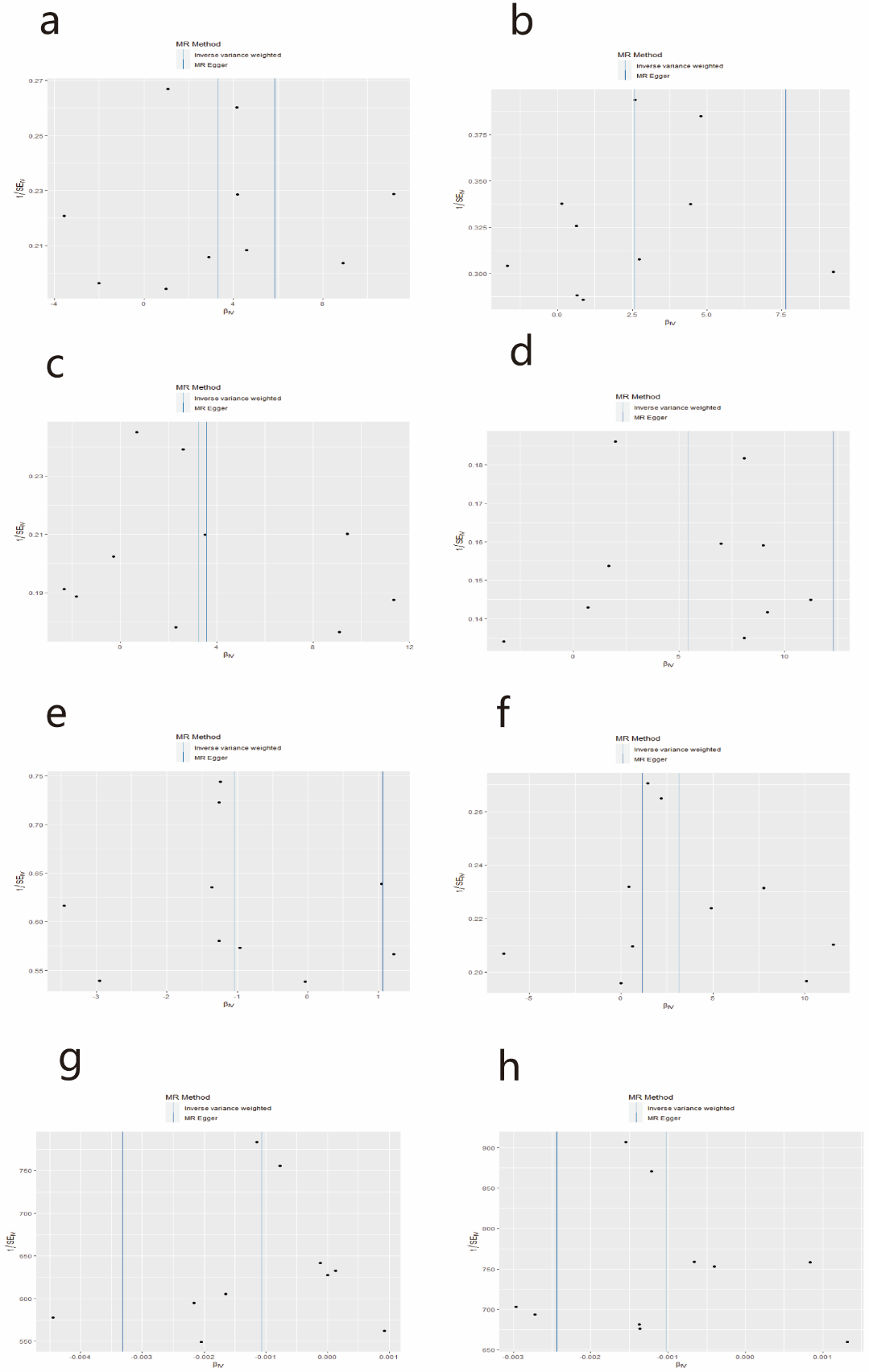
**

**Figure S7.** Scatter plots of significant estimates from genetically predicted fibrosis stage on (a) without global weighted TH of the isthmuscingulate; (b) with global weighted TH of the isthmuscingulate.

**
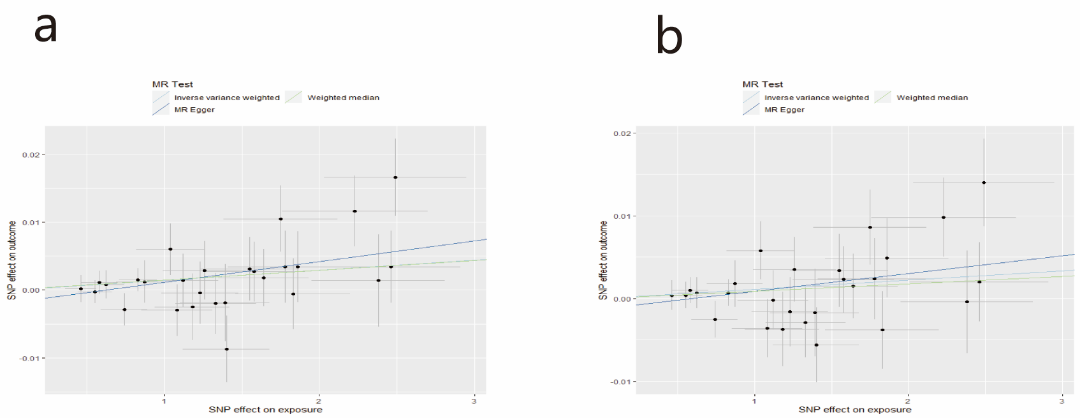
**

**Figure S8.** Leave-one-out plots of significant estimates from genetically predicted fibrosis stage on (a) without global weighted TH of the isthmuscingulate; (b) with global weighted TH of the isthmuscingulate.

**
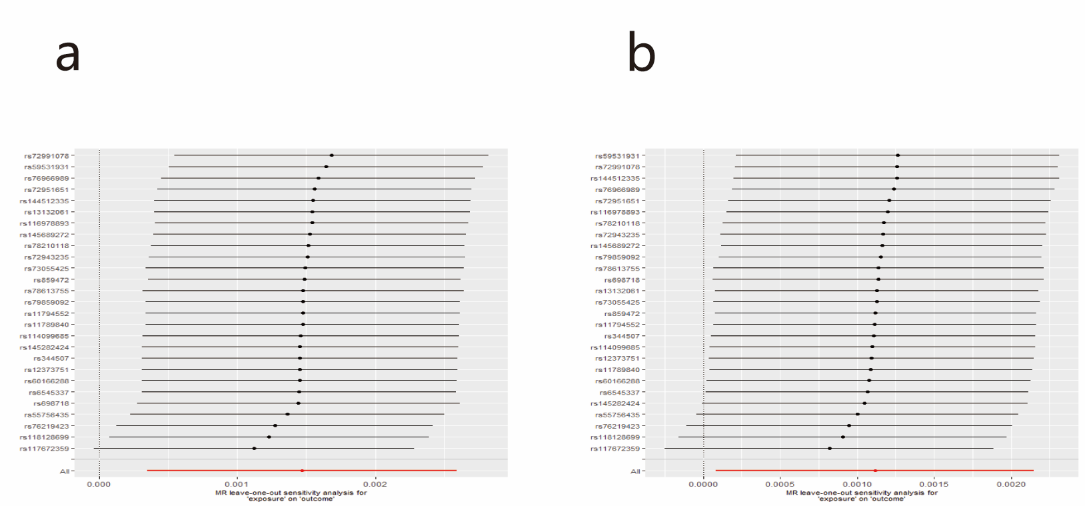
**

**Figure S9.** Funnel plots of significant estimates from genetically predicted fibrosis stage on (a) without global weighted TH of the isthmuscingulate; (b) with global weighted TH of the isthmuscingulate.

**
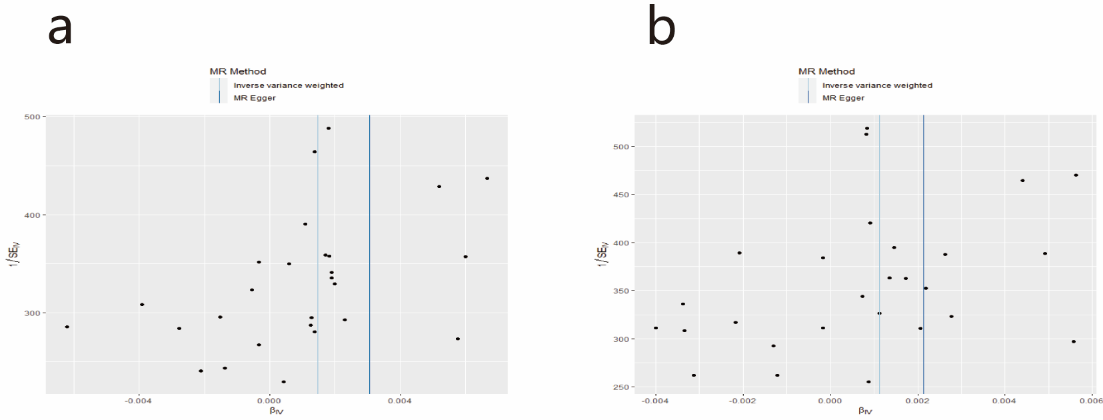
**
